# Race and gender representation of hypertrophic cardiomyopathy or long QT syndrome cases in a South African research setting

**Published:** 2007-07

**Authors:** M Heradien, A Goosen, PA Brink, JC Moolman-Smook

**Affiliations:** Department of Internal Medicine, University of Stellenbosch, Stellenbosch; Department of Internal Medicine, University of Stellenbosch, Stellenbosch; Department of Internal Medicine, University of Stellenbosch, Stellenbosch; US/MRC Centre for Molecular and Cellular Biology, University of Stellenbosch, Stellenbosch

## Abstract

**Summary:**

We researched hypertrophic cardiomyopathy (HCM) and long QT syndrome (LQTS) as models for studying the pathophysiology of arrhythmias and hypertrophy, and in the process we have had the opportunity to compare local disease profiles with global patterns.

We trawled our database entries over the past 20 years to identify all cases of heart muscle and arrhythmic disease. Among these, we separated the index cases from the rest of their family members, segregating for the relevant heart disease, so that numbers were not biased by family size, and analysed the race and gender composition of the HCM and LQTS sectors.

The majority of HCM index cases (*n* = 90, 51.1% of HCM index cases) were of mixed ancestry (MA), with white Caucasian ancestry following closely behind with 74 cases (42.0%); only a few black African (*n* = 9, 5.1%) or Indian/Asian (*n* = 3, 1.7%) cases were seen or referred. The LQTS index cases were almost exclusively white Caucasian (*n* = 36, 88% of LQTS index cases), with four cases (9.8%) of MA, one (2.4%) of Indian/Asian and none of black African descent. These race demographics did not fit the national demographics for South Africa as a whole. In contrast, in both groups, gender biases (slightly more male than female HCM cases, and a 0.4 ratio of males to females in LQTS) previously reported elsewhere appeared to be replicated in our database.

Genetic bias is an unlikely explanation for the skewed demographics in our database; a more likely explanation relates to various missed opportunities to diagnose, missed diagnoses and misdiagnoses, as well as the real population drainage of our main referral centre in the context of a differentiated healthcare system.

## Summary

Over the last 20 years, scientific endeavours aimed at mapping Mendelian inherited disease to chromosomal segments often uncovered causal genes of an unanticipated nature. These unpredicted genes pointed to and paved the way for the exploration of novel pathological mechanisms leading to morbidity and death, the understanding of which, potentially, could also benefit sufferers from common disease, as similar pathways may be at play in both the common complex and rarer inherited diseases.^1^ A now-classic example of the benefits of understanding the pathophysiology of inherited disease, which even predates the molecular era, is that of studies of familial hypercholesterolaemia (FH), an autosomal dominantly inherited disease. These studies lead to the identification of the key role that the enzyme 3-hydroxy-3-methylglutaryl coenzyme A reductase (HMGCoA) plays in the control of intracellular cholesterol synthesis and the current widespread use of ‘statin’ drugs.^2^,^3^

Our molecular genetic research has, for the last two decades, focused on inherited heart disease, primarily hypertrophic cardiomyopathy (HCM), the long QT syndrome (LQTS) and familial conduction disease. In a family context, these diseases are very often asymptomatic, but with the risk of syncope as the result of an arrhythmia, or more dire, death, during an arrhythmic event. Analogous to the FH example, we investigated these diseases as models for studying the pathophysiology of two phenomena, namely, arrhythmias and hypertrophy, which are common to a variety of cardiac conditions. In the process, we have had the opportunity to compare local disease profiles with global patterns.

In this regard, we find it intriguing that, although the gender distribution for HCM and LQTS in our patient database was in accordance with international male:female ratios for these diseases, the racial distribution was not representative of national demographics. We describe the extent of this lack of representation and speculate on the likely reasons. The conduction diseases are excluded, as they have been uniquely described in this country, and cannot be meaningfully compared with reports elsewhere.

## Methods

Demographic details of patients with cardiac conditions are routinely entered into our patient database at their referral for genetic investigations by our laboratory. Diagnosis of LQTS^4^ and HCM^5^ were made by criteria previously reported.

We trawled this database to identify all cases of heart muscle and arrhythmic disease. Among these, we separated the index cases from the rest of their family members, segregating for the relevant heart disease so that numbers were not biased by family size, and analysed the race and gender composition of the HCM and LQTS sectors.

In order to ascertain the frequency of HCM and LQTS reported from Africa, a MEDLINE search was performed with the search terms ‘hypertrophic cardiomyopathy’ and ‘Africa’. The same search was repeated but using all African country names individually. A similar search was conducted for the long QT syndrome or its eponym, namely, ‘Romano-Ward’ or ‘Ward-Romano’ syndrome.

## Results and discussion

A total of 743 cases (286 index cases) of various heart, heart muscle and conduction diseases were seen or referred and entered into the database during the period 1986 to 2006. Of these 371 (176 index cases, 62% of index cases) had hypertrophic cardiomyopathy and 246 (41 index cases, 14% of index cases) had LQTS.

The majority of HCM index cases (*n* = 90, 51.1% of HCM index cases) were of mixed ancestry (MA), with white Caucasian ancestry following closely behind with 74 cases (42.0%). Very few black African (*n* = 9, 5.1%) or Indian/Asian (*n* = 3, 1.7%) cases were seen or referred (Table 1). The LQTS index cases were almost exclusively white Caucasian (*n* = 36, 88% of LQTS index cases), with four cases (9.8%) of MA, one (2.4%) of Indian/Asian and none of black African descent (Table 2). National demographics for South Africa as a whole (42 million people) are given as 35.4 million (79%) black Africans, 4.2 million (9.6%) white Caucasian, 4 million (8.9%) of mixed ancestry and 1.1 million (2.5%) Indian/Asian.^6^

**Table 1:** Race And Gender Distribution Of Hypertrophic Cardiomyopathy Index Cases

*Ancestry*	*Number*	*% of total*	*F*	*M*	*M:F*
Black	9	5.1	3	6	2.0
Indian/Asian	3	1.7	1	2	2.0
MA	90	51.1	42	48	1.1
White	74	42.0	20	54	2.7
Total	176	100	66	110	1.7

F = female, M = male, M:= 5 male-to-female ratio, MA = mixed ancestry.

**Table 2:** Race And Gender Distribution Of Long Qt Syndrome Index Cases

*Ancestry*	*Number*	*% of total*	*F*	*M*	*M:F*
Black		0			
Indian/Asian	1	2.4	1	-	-
MA	4	9.8	2	2	-
White	36	87.8	26	10	0.4
Total	41	100	29	12	0.4

F = female, M = male, M:F = male-to-female ratio, MA = mixed ancestry.

Gender breakdown in the HCM index case group was 110 males (62.5%) versus 66 females (37.5%), whereas in the LQTS group, 12 patients (29.3%) were male, compared to 29 (71.7%) females. In the HCM group, the male-to-female ratio varied from 1.1 in MA, to 2.0 in black and Indian/Asian, and 2.7 in white patients (Table 1). In LQTS, a male-to-female ratio of 0.4 was maintained in the white and MA groups. The ratio could not be established for black and Indian/Asian patients due to low numbers (Table 2). Therefore, in both groups, gender biases (more male than female HCM cases,^7^,^8^ and a 0.4 ratio of males to females in LQTS) previously reported elsewhere appeared to be replicated in our database.^9^

However, although gender demographics were maintained for both diseases, the question remains why very few persons of black African descent with HCM and none with LQTS appeared in our database. HCM in black individuals has been infrequently reported from South Africa^10^-^12^ and more recently, in two reports, also from other African countries,^13^,^14^ although HCM was found to be more than twice as prevalent in black Americans as in white Americans in a prospective study.^7^

For LQTS, where the diagnosis is made on syncope and a prolonged QT interval on an ECG, not a single case report from Africa can be traced in the literature, despite the long-standing and common availability of the diagnostic technology. Those reports from Africa on QT variation pertain to conditions other than LQTS, such as kidney failure,^15^ HIV/AIDS,^16^ eclampsia,^17^ Kwashiorkor^18^ and treatment of malaria with halofantrine.^19^ Yet, the ratio of black American to white American LQTS patients was almost 1.5:1, and that of Asian American to white American almost 1:1 in a panel of patients referred for genetic screening in the USA.^20^ The paucity of reports of HCM and LQT from Africa begs an explanation, and may relate to the genetics or the clinical diagnoses of these inherited cardiac conditions.

The prevalence of Mendelian inherited disease, implying causal genetic mutations with strong pathological effects could become racially restricted for a variety of reasons, such as genetic drift, mutation rates and selection. For example, myotonic dystrophy is a disease that is, barring a single report, not reported from Africa, but is observed more frequently in other areas of the world. An explanation for this phenomenon has been based on the absence of a single ancestral mutation-predisposing genetic state (pre-mutation) of a chromosomal segment in African populations, while this same pre-mutation is present in populations elsewhere that derived from the African diaspora.^21^,^22^ This may represent an instance of genetic drift, where the pre-mutation simply drifted into non-existence in the African populations. An alternative explanation is that the mutation might only have occurred in the migrant population from which the rest of the world population then derived.

This scenario is extremely unlikely for both HCM and LQTS, as many *de novo* mutations have been described for both. For hypertrophic cardiomyopathy, more that 400 mutations in 12 different genes, located on different chromosomal regions, have been described,^23^ and similarly, for LQTS more than 400 mutations in eight differentially located genes^24^ exist. The disease-causing mutations are most often ‘private’, meaning that such genetic defects generally arise independently in each given family, even when mutations appear to be shared, and so will not be associated with any particular allelic chromosomal segment in a population. Also, as would be expected of disease alleles, no increase to the level of polymorphic frequencies through positive selection or through drift has been reported. Therefore, for both diseases, individuals carrying disease-causing mutations should be present in all population groups to the same extent; however, this is clearly not the case in our patient sample.

One possible explanation revolves around penetrance of the diseases, that is, the probability that an individual carrying a disease-causing mutation will manifest the disease clinically. Both diseases are characterised by variability of presentation, even within family members bearing the same mutation, supporting the notion of additional modifying environmental or genetic factors influencing the final disease picture. If modifiers of either type, which on one hand exacerbate disease severity such that early death, an example of selection, removes such individuals from the population, or, on the other hand, ameliorate disease presentation to asymptomatic longevity, are particularly prevalent in some populations, disease demographics would be skewed. However, the racial demographics from overseas studies mentioned earlier suggest that this is an unlikely scenario.

We therefore need to question whether other selection biases, such as identification bias, may be operating. Certainly, the fact that many of our patient base reported to Tygerberg Hospital, which primarily drained traditionally white and MA sectors of the Cape metropole is a factor. However, our presence in the Western Cape at a historically white university is unlikely to be the only explanation. Our work has been vigorously propounded countrywide, through active exposure at local academic meetings, public talks and the media and direct mass mailing to clinicians. We have, for example, in recent years mailed more than 2 000 letters to physicians and cardiologists across the country, explaining our LQTS research and requesting participation. Therefore, barring apathy on the part of potentially participating clinicians or patients, one might still draw the conclusion that the prevalence of manifest HCM and LQT differs among our subpopulations.

Another, and in our opinion, the most likely reason for the phenomenon may relate to a recognition bias, and such recognition biases may have multiple causes, involving both missed opportunities to diagnose (because the patient never comes to clinical attention), missed diagnoses and misdiagnoses.

Both HCM and LQTS diagnoses depend on the availability of equipment, mainly echocardiography for HCM and ECG for LQTS, utilisation of this equipment and the interpretation of the tests performed with such equipment. In HCM, where patients are often asymptomatic or mildly affected and symptomatic, referral for diagnostic echocardiographic investigation often springs from clinical identification of a systolic murmur. Echocardiography is less widely available than, for instance, ECG, and certainly, in the past the paucity of reporting of this disease from Africa has been attributed to unavailability of echocardiography.^25^,^26^ However, this is no longer likely to be the limiting factor in Africa’s big cities. On the other hand, echocardiography is more time consuming and dependent on specialised skills, and such constraints in the context of other overwhelming healthcare priorities in a primary-care setting may lead to lower referral rates and consequently missed opportunities for diagnosis.

In LQTS on the other hand, ECG availability and interpretation are not generally problematical; rather, interpretation of episodes of transient loss of consciousness (TLOC) may result in misdiagnoses. If, for example, an episode of TLOC is regarded as epilepsy, ECG is conventionally simply not performed. The correct interpretation of TLOC derives from awareness among clinicians of the correct questions to ask in order to obtain a differentiating case history, and awareness among the public about the difference between ‘fits’ and other causes of TLOC.^27^ It is very disconcerting that the same graduate students who pass or fail examinations on their ability to identify murmurs, are never examined on their ability to differentiate TLOC. Certainly, the extent of misdiagnoses in LQTS is substantial. When we started researching LQTS in 1992, more than 80% of diagnosed LQTS patients had at some previous stage been diagnosed, and often treated, as epileptics.^28^

We must accept that in both hypertrophic cardiomyopathy and in the long QT syndrome, opportunities to diagnose such patients may not present themselves and when they do present, the diagnosis may be missed. A likely reason is that in a country where premature death most often occurs in obviously ill persons, most often suffering from infectious disease,^29^ diseases in those more apparently well may simply go undiagnosed, even when such diseases convey a high risk of death, such as HCM and LQT. A much reported case from the Cameroon, Africa, has been that of Marc Vivian Foë, a world-class soccer player, where the diagnosis of HCM was made only after he collapsed and died on a soccer field in France.^30^ In the time- and manpower-pressured setting of primary healthcare clinics in Africa, a less sophisticated service and a language barrier during translation of questions and answers may lead to loss of diagnostic subtleties, whereas the same constraints may not apply to private healthcare settings. Therefore the bias in our database may be a combined reflection of the real population drainage of our main referral centre (Tygerberg Hospital) and the consequence of a differentiated healthcare system nationwide.

## Conclusions

The most plausible reasons for demographic differences in the prevalence of HCM and LQTS in the South African population as reflected in our database probably relate to various missed opportunities to diagnose, missed diagnoses and misdiagnoses in the context of a differentiated healthcare system. With a high emphasis on persons with obvious physical disease, diseases that often manifest as less chronic and less severe are simply not diagnosed. Yet, the medical and lay community should be made aware of such diseases as young lives could be saved by the timely institution of appropriate therapy.
